# Investigating inlay designs of class II cavity with deep margin elevation using finite element method

**DOI:** 10.1186/s12903-021-01630-z

**Published:** 2021-05-16

**Authors:** Yung-Chung Chen, Chi-Lun Lin, Chun-Hsien Hou

**Affiliations:** 1 School of Dentistry and Institute of Oral Medicine, Medical College, National Cheng Kung University, Tainan, Taiwan, Republic of China; 2 Division of Prosthodontics, Department of Stomatology, National Cheng Kung University Hospital, National Cheng Kung University, Tainan, Taiwan, Republic of China; 3 Department of Mechanical Engineering, National Cheng Kung University, Tainan, Taiwan, Republic of China

**Keywords:** Finite element analysis, Dental cavity preparations, Dental inlay

## Abstract

**Background:**

This study evaluates the mechanical performance of deep margin elevation technique for carious cavities by considering the shape designs and material selections of inlay using a computational approach combined with the design of experiments method. The goal is to understand the effects of the design parameters on the deep margin elevation technique and provide design guidelines from the biomechanics perspective.

**Methods:**

Seven geometric design parameters for defining an inlay’s shape of a premolar were specified, and the influence of cavity shape and material selection on the overall stress distribution was investigated via automated modelling. Material selection included composite resin, ceramic, and lithium disilicate. Finite element analysis was performed to evaluate the mechanical behavior of the tooth and inlay under a compressive load. Next, the analysis of variance was conducted to identify the parameters with a significant effect on the stress occurred in the materials. Finally, the response surface method was used to analyze the stress responses of the restored tooth with different design parameters.

**Results:**

The restored tooth with a larger isthmus width demonstrated superior mechanical performance in all three types of inlay materials, while the influence of other design parameters varied with the inlay material selection. The height of the deep margin elevation layer insignificantly affected the mechanical performance of the restored tooth.

**Conclusions:**

A proper geometric design of inlay enhances the mechanical performance of the restored tooth and could require less volume of the natural dentin to be excavated. Furthermore, under the loading conditions evaluated in this study, the deep margin elevation layer did not extensively affect the strength of the tooth structure.

**Supplementary Information:**

The online version contains supplementary material available at 10.1186/s12903-021-01630-z.

## Background

Deep margin [1] is the phenomenon whereby, because of deep caries or severe structural defects, a cavity is lower than the gingival margin after removing caries or unsound dentin. The issue usually occurred at the proximal surface of a tooth so that it is difficult to be detected at an early stage. Clinically, the deep margin causes difficulties in isolating the infected area from oral fluids, such as blood, saliva, and gingival sulcus fluid, before applying restorative materials. Inadequate isolation can cause contamination during the application of dental adhesives, which may reduce the strength of the adhesive. Moreover, when the indirect restoration is indicated, the marginal accuracy of the impression can also be affected. Therefore, for a cavity with a deep margin, making the margin accessible above the gingiva before performing restorative procedures is necessary.

The most common clinical method for solving the problems related to the deep margin is the crown lengthening procedure, whereby the gingival margin of the cavity is directly exposed through periodontal surgery. The drawback of this technique is the reduced crown-to-root ratio of the tooth. An unfavorable crown-to-root ratio causes the tooth vulnerable to the occlusal force and poor mechanical performance of the prosthesis, which could further lead to prosthetic failure.

Another proposed solution is deep margin elevation (DME). In 1998, Dietschi and Spreafico [2] proposed a filling method to improve the bonding of an indirect restoration with the subgingival margin. The method used composite resin to fill the cavity floor beneath the gingiva to render the margin visible above the gingiva for the fabrication of an indirect restoration. The method was originally termed the Sandwich Technique [3]; when applied to proximal cavities, but later has also been referred to as the Proximal Box Elevation (PBE). In 2012, Magne [1] again proposed using the composite resin to fill subgingival margins as an alternative to the crown lengthening procedure and termed this technique as DME. This technique could maintain crown-to-root ratio, prevent unfavorable effects on tooth stability, and reduce postoperative healing time and complications; the cost was also lower than that of the crown-lengthening procedure.

Glass ionomer cement (GIC) was initially used as the filling material for DME. Because of its brittleness [4] and lower mechanical strength, the load-bearing performance was unfavorable [5, 6]. During the past decades, the flowable resin has gradually gained recognition in DME because of its improved durability. Frankenberger et al. [7] prepared a mesial-occlusal-distal (MOD) cavity in the third molar, whereby the cavity’s depth was set to 2–3 mm below the cementoenamel junction (CEJ). Two techniques of composite resin filling were used to elevate the floor: filling the cavity without layering or with three 1-mm thick layers. After completing PBE, the cavity was filled with a ceramic inlay and through thermal-load cycling. Finally, scanning electron microscope (SEM) examination revealed that the control group in which ceramic inlay directly bonded to dentin without PBE achieved the highest gap-free margin percentage (92% Gap-free). However, the samples filled using three-layered composite resin also achieved close to an 84% gap-free margin, which is sufficient for replacing the direct bonding of ceramic and dentin. Zaruba et al. [8] prepared a MOD cavity in forty human molars and divided them into four groups. The cavity-floor margin of Group 1 was located 1 mm above CEJ, and the inlay restoration was ceramic; the cavity floor margins of other groups were set at 2 mm below CEJ. The inlay restoration in Group 2 was ceramic at the upper part and 3-mm-thick composite resin at the lower part. The inlay restoration in Group 3 was ceramic at the upper part and two layers of 1.5-mm-thick composite resin at the lower part. The inlay restoration in Group 4 was fully ceramic. The SEM results revealed that whether the composite resin was used or not, the integrity of the margin bonded to the dentin was the same. Ilgenstein et al. [9] prepared a MOD cavity over mandibular molars after root canal therapy by placing the distal proximal box 2 mm below the CEJ. The samples were divided into four groups: PBE with ceramic restoration; PBE with composite resin restoration; ceramic restoration only; and composite restoration only. The results revealed no significant differences between Groups 1–3, and although Group 4 exhibited an enhanced margin quality, the average fracture value was high. These results demonstrated that PBE did not affect the integrity or fracture behavior of the margins of mandibular molars with ceramic restoration after root-canal treatment.

From the standpoint of increasing restoration's resistance, several studies have been proposed and investigated [10–13]. The principles of minimally invasive dentistry suggest preparing the cavity as conservative as possible; however, much less invasive cavity preparation could be disadvantageous for the stability of the restorations. A partial coverage crown can provide higher fracture strength than an inlay for the post-endodontically treated cavities [10]. Among those common bonded partial restorations, an onlay requires less tooth preparation than a partial crown and provides more cuspal coverage than an inlay. But more details regarding onlay design, such as existence of pulpal extension, need to be considered [11]. Moreover, material selection can counteract the benefit of extensive structural coverage [12]. Although onlay coverage seems to provide greater fracture resistance, the tooth usually cannot be restored again once the restoration fails [13]. Therefore, long-term stability of a restoration depends on various factors, including the material used, individual’s occlusal force, and remaining tooth structure [10–13]. Finite element analysis becomes the best tool for seeking the optimum design and most results demonstrated a correlation between the geometry of the restoration and the stress distribution of the restored tooth [14–20].

The past studies have only discussed a few individual geometric configurations of inlay designs. Studies related to DME have mostly been limited to inspect margin integrity after restoration; the effect of multi-material restoration on mechanical behavior has not yet been comprehensively studied. With proper isolation and moisture control, the bonding procedure of DME should be able to perform smoothly. In addition, the crown lengthening procedure usually results in a worse crown-to-root ratio which can further affect the structural stability [18]. The mechanical performance for the whole entity, restoration, and tooth, can be further enhanced via the approach of shape optimization favored by engineering design.

This study aimed to seek ideal layouts of inlays for three mainstream restorative materials after using DME to restore the cavity’s floor based on the finite element method. The design space of cavity shape was assembled with several geometric parameters. The effect of the cavity shape and material selection on overall stress distribution was investigated.

## Methods

### Finite element modeling

A human first premolar was scanned using a Micro-CT scanner (Skyscan 1076, Bruker Corp., Belgium, scanned using voxel size of 0.127 mm and 720 image slices) to obtain DICOM images. The medical image processing software (Mimics 16.0, Materialise, USA) was used to construct the three-dimensional surface of the premolar (STL file format). The premolar model then underwent post-processing through computer-aided-design software (Geomagic 12, 3D Systems, USA) to produce a three-dimensional geometric model.

On the premolar model, a cavity containing an occlusal valley with an open proximal box was designed, and a class II inlay and a DME layer were created using the finite element analysis software (ANSYS 15.0, ANSYS Inc., USA). The premolar was placed into a cylindrical bone block with a top layer of 2 mm thick as cortical bone and the remainder as cancellous bone. The final model contained six components: enamel, dentin, inlay, DME layer, cortical bone, and cancellous bone. Three types of inlay material were considered, including composite resin (CO), ceramic (CE), and lithium disilicate (LD). The material of the DME layer was fixed as a type of flowable resin. The material of all components in the finite element model were assumed to be linearly elastic, homogeneous, and isotropic, and their Young’s moduli and Poisson’s Ratios were [18.6 GPa, 0.31] for dentin, [84.1 GPa, 0.33] for enamel, [13.7 GPa, 0.3] for cortical bone, [1.37 GPa, 0.31] for cancellous bone, [15 GPa, 0.35] for CO inlay, [45 GPa, 0.25] for CE inlay, [90 GPa, 0.25] for LD inlay, [5 GPa, 0.35] for DME layer. [14, 15, 21–26].

**Fig. 1:**
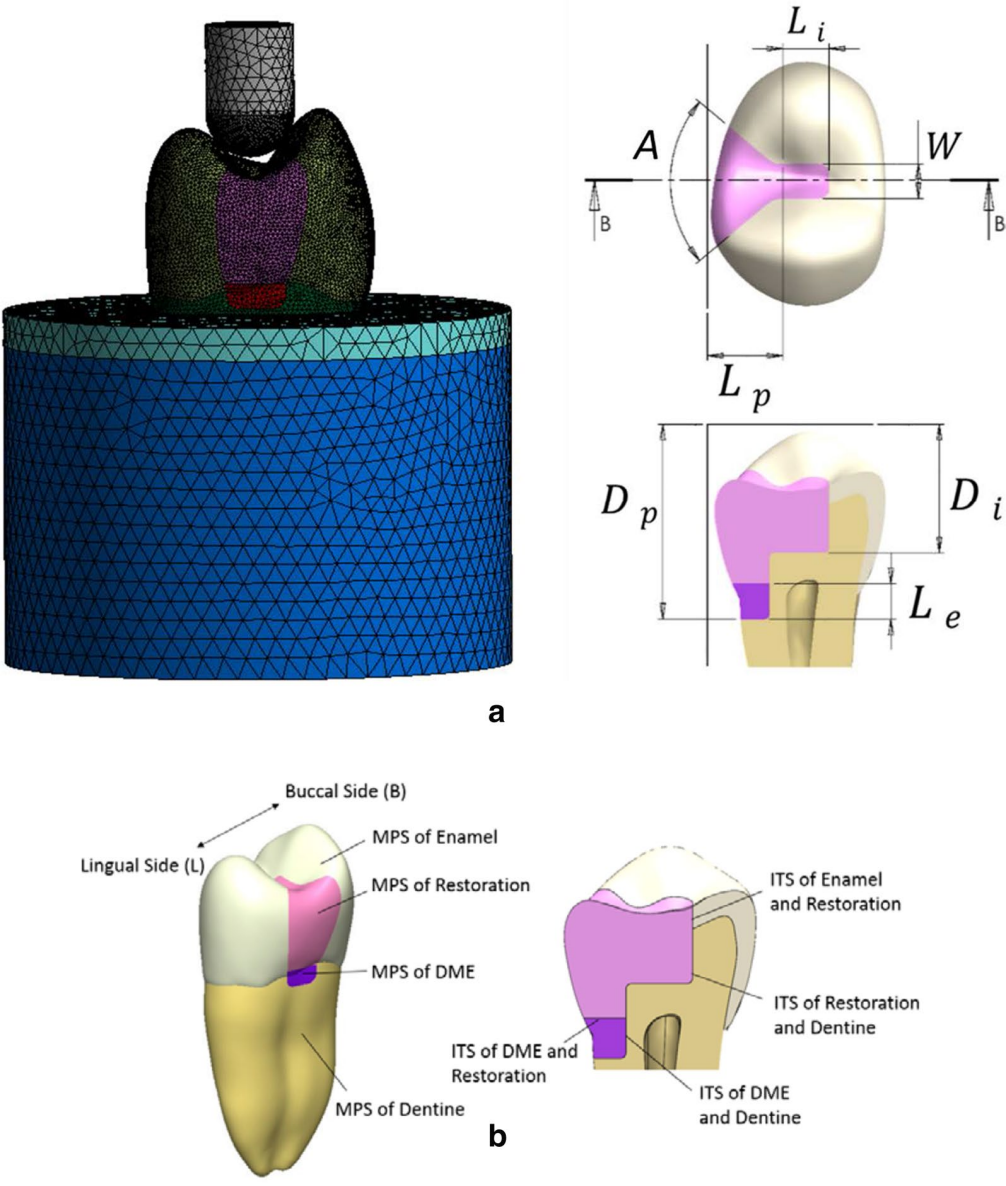
**a** The left-hand side shows the finite element model with meshes, where the geometric parts are in different colors. The right-hand side displays two views of the tooth with definitions of the seven design parameters. **b** The eight stress indexes evaluated, which include two types of stresses, MPS and ITS

The mesh used 10-node tetrahedral structural solid (Solid 187) elements. A study of mesh convergence was performed by evaluating the mesh size between 0.15 and 0.7 mm to ensure the accuracy of the numerical results. The convergence was found at the mesh size smaller than 0.22 mm, but the computation time would greatly increase when the mesh size was smaller than 0.2 mm. Therefore, the global mesh size was set as 0.2 mm for optimizing both numerical accuracy and computation cost, and it resulted in approximately 435,500 nodes and 265,900 elements for the entire model (where the numbers of nodes and elements of individual parts are 152,682 and 90,653 for dentin, 86,331 and 49,580 for enamel, 19,140 and 10,869 for the restoration, 2,534 and 1,335 for resin, 22,477 and 13,488 for cortical bone, and 126,372 and 83,547 for cancellous bone. Note that the numbers slightly varied in different design configurations). A 4-mm–diameter round indenter applied a compressive load of 600 N to the premolar, parallel to the long axis of the tooth. In general, 100–800 N of biting forces can be measured from healthy persons and patients of various muscle efficiencies [27]. To proper simulate the biting force, a 600 N load which has been used for the premolar in a numerical study [28] was chosen in the present study. Before simulating the compressive loading, the indenter was centered to the long axis of the tooth with a short distance away from the tooth top and simulated to move downward until contacting the tooth. This pre-simulation step was to find the realistic contact condition between the tooth and the indenter, which consequently occurred on the premolar at two points (located at the buccal and lingual slopes of the occlusal surface, respectively). The bottom of the bone block was constrained in all directions (Fig. 1). Since this study focused on the inlay design, cement failure is not of concern. Therefore, the dental restoration cement layer was not modeled and the contacts between materials in the model were assigned perfect bounding conditions. This simplification is reasonable and well accepted in the literature [37–39]. The quasi-static stress analysis with ANSYS was carried out to evaluate the inlay designs. Seven design parameters and the considered design space were defined as follows, the width of isthmus (*W*, 1.6 mm–2.4 mm), angle of divergence (*A*, 85°–120°), length of isthmus (*L*_*i*_, 1–2 mm), length of proximal box (*L*_*p*_, 2.3–3 mm), depth of isthmus (*D*_*i*_, 4.6–6 mm, measured from the highest point of the tooth to the cavity location), depth of proximal box (*D*_*p*_, 7–9 mm), and elevation height (*L*_*e*_, 0.35–1 mm). Note that these measurements were not directly from the tooth surface (see Fig. 1a), so it should not lead to any pulpal damage.

### Validation experiment

**Fig. 2:**
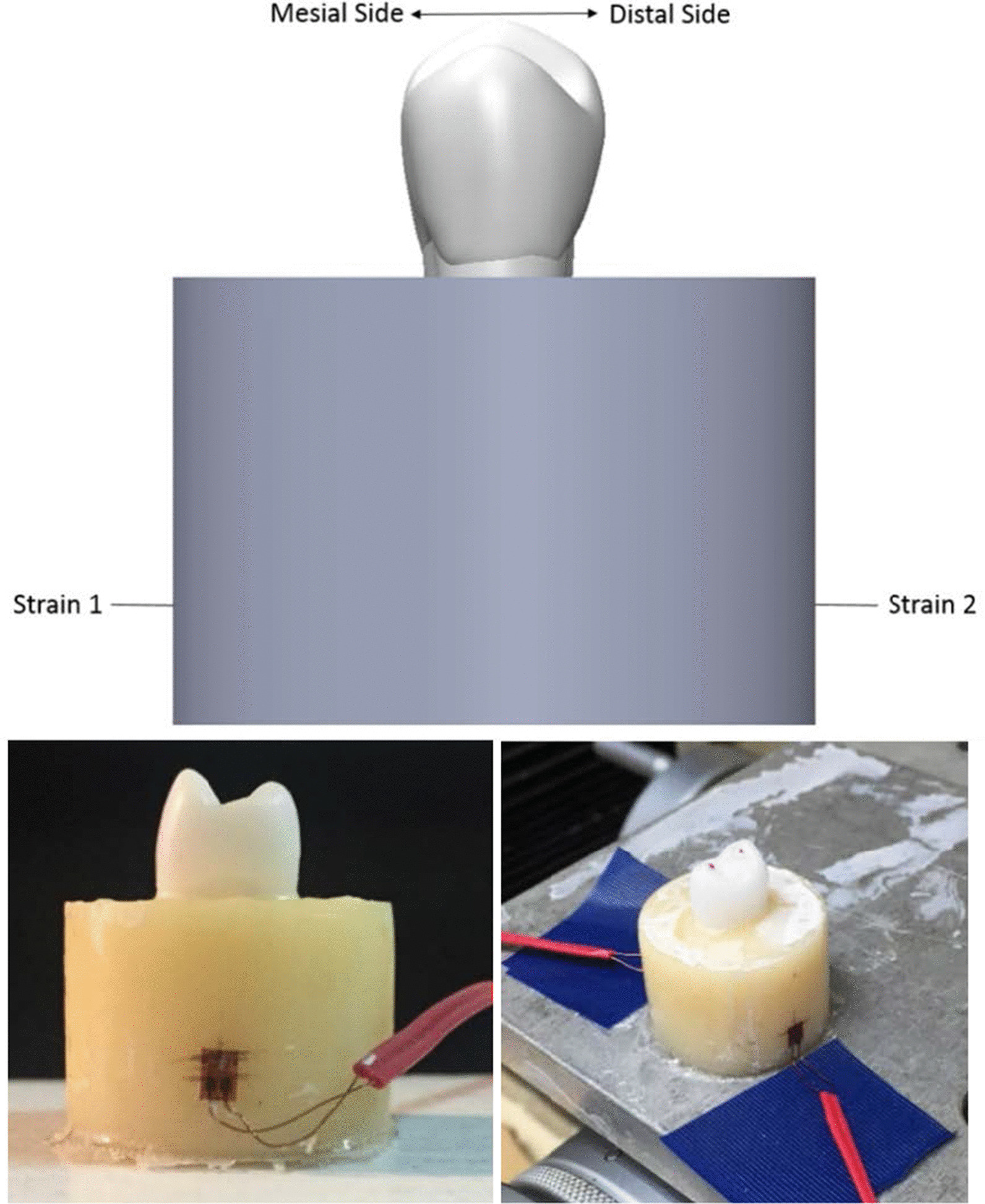
An illustration of validation experiment model and the placements of strain gauges is shown on the top. The two photos on the bottom show the real setup

Compression tests were conducted (Fig. 2). The materials of the specimen were CNC-milled zirconia (VITA YZ, VITA Zahnfabrik, Germany) for the tooth model (210 GPa) and PMMA (VITA CAD-Temp®, VITA Zahnfabrik, Germany) for the cylindrical bone blocks (2.8 GPa). An adhesive (3 M U200, 3 M, USA) was applied to bond the tooth model and bone block. The specimen was tested in a universal testing machine (AG-I, Shimadzu Corp., Japan) at 2 N/s until 400 N (a moderate human bite force was chosen) was achieved, which was then maintained for 60 s. Strain gauges (KFGS-02-120-C1-11, Kyowa Electronic Instruments Co., Ltd. Japan) were attached to the mesial side (Strain 1) and distal side (Strain 2) of the bone block. The data-acquisition system, including a 4-slot USB chassis (cDAQ-9174, National Instruments, USA) and an 8-channel capture module (NI-9235, National Instruments, USA), was used to record the strain values generated during the loading. The measured strains were compared with the results of a finite element simulation that used the same experimental setting.

### Mechanical analysis for design suggestions

Eight stress indexes (Fig. 1b) were retrieved from the results of stress analysis to assess the mechanical performance of the tooth and inlay restoration. Two types of stress items were obtained, which were peak interfacial tensile stress (ITS) at the various interfaces, and peak maximum principal stress (MPS) in all components of the tooth. Four peak ITS items (on the right-hand side of Fig. 1b) were obtained by retrieving the most negative value of the contact pressure at the interface between each contact pair of materials that were of interests. Four peak MPS items were the highest values of MPS found in each selected material component (on the left-hand side of Fig. 1b). An analysis of variance was conducted to assess the influence of each of the seven design parameters on each stress index and calculate the main effect. The design parameters that demonstrated a significant influence on each stress index were identified, and a response surface of each stress index to those critical design parameters was established through Latin Hyper Cube Sampling. By assessing these response surfaces, guidelines were proposed for design choices to enhance the mechanical performance of the tooth and inlay restoration.

## Results

### Validation results

The mean differences between the experimental results measured by the two strain gauges (− 110.4 and − 32.8 micro-strain) and the simulation results (− 103.0 and − 31.1 micro-strain) were 7.18% and 6.06%, respectively. The standard deviations of the strain values obtained from the two strain gauges (Strain gauges No. 1 and 2) in the validation experiment were approximately 2.9 and 1.6 micro-strain, respectively. Given the accuracy of the hardware module is 2.7 micro-strain, the measured values were within a reasonable range. Therefore, the experimental validation revealed that the finite element model could provide a sufficiently accurate prediction.

### Main effect analysis

For the CO, CE, and LD inlays, the peak MPS of enamel was primarily affected by *L*_*i*_ and *L*_*p*_, while the effect of *W* on the peak MPS increased as the increase of Young’s modulus of the inlay. The peak MPS of each inlay material was mainly affected by *L*_*i*_ and *L*_*p*_. Although the peak MPS of the DME layer was also significantly affected by a few parameters, their values in all three cases fell in the range of 1–7 MPa, which is far less than the tensile strength of the commonly used flowable resin (42 MPa) [31]; therefore, no further discussion was needed. The peak MPS in dentin was not significantly influenced by any parameter. The relationship between each parameter and the peak MPS in different layers is displayed in Additional File 1.

The peak ITS of the inlay to enamel and dentin were separately investigated on the buccal side and lingual side. On the buccal side, the ITS of all three types of inlay material to enamel was primarily influenced by *W*. On the lingual side, the variation of peak ITS for CO inlay to enamel was small, while the peak ITS of inlay to enamel was significantly influenced by L_*i*_, L_*p*_*,* and *W* for CE and LD inlays. The peak ITS on the inlay-dentin interface was only significantly affected on the lingual side for all three types of inlay materials. The relationship between each parameter to peak ITS is as displayed in Additional File 2.

Although the peak value of ITS on the other interfaces was also subject to parameter influence, the peak value change due to the variation of parameters within the design space was small and did not affect the risk of material damage.

### Response surface analysis

In this section, we discuss the responses of MPS and ITS to their high-impact design parameters.

#### Peak MPS in the enamel and inlay

**Fig. 3:**
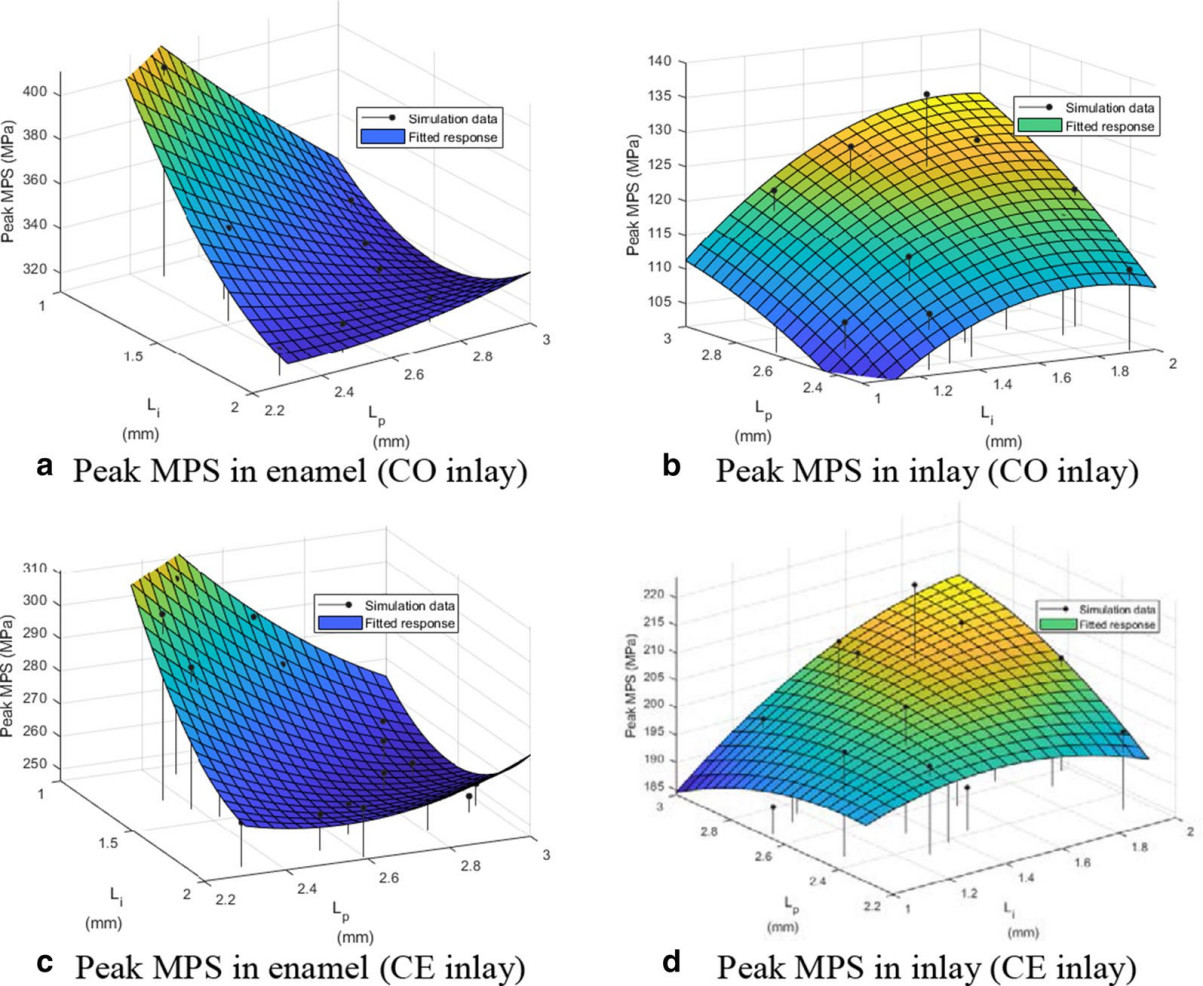
The response surfaces of peak MPS in enamel and inlay to L_i_ and L_p_

For the CO inlay case, the response surface of the peak MPS in the enamel corresponding to *L*_*i*_ and *L*_*p*_ is presented in Fig. 3a. The simulated peak MPS value was between 316.45 and 405.94 MPa. The root–mean–square error (RMSE) between the predicted values provided by the response surface and simulation was 1.29%. The response surface of the peak MPS in the CO inlay corresponding to *L*_*i*_ and *L*_*p*_ was shown in Fig. 3b. The peak MPS was between 106.63 and 135.06 MPa with an RMSE of 3.20%.

For the CE inlay case, Fig. 3c presents the response surface of the peak MPS in the enamel corresponding to *L*_*i*_ and *L*_*p*_. The peak MPS ranged from 251.36 to 306.16 MPa, with an RMSE of 5.98%. The response surface of the peak MPS in the CE inlay corresponding to *L*_*i*_ and *L*_*p*_ is shown in Fig. 3d. The peak MPS value was between 189.11 and 218.77 MPa, with an RMSE of 12.66%.

**Fig. 4:**
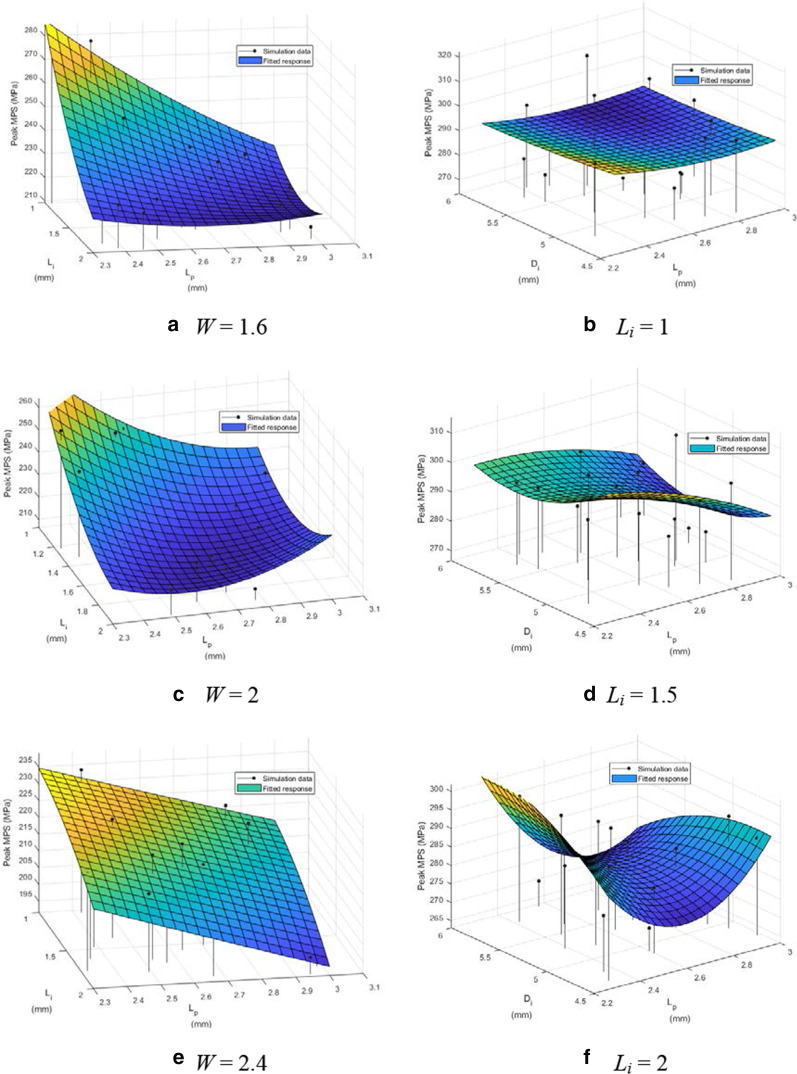
The response surfaces in column 1 are the peak MPS in enamel to *L*_*i*_ and *L*_*p*_, plotted at *W* = 1.6, 2, and 2.4 mm. The response surfaces in column 2 are the peak MPS in inlay to *L*_*p*_ and *D*_*i*_, plotted at *L*_*i*_ = 1, 1.5, and 2 mm. (LD inlay)

For the LD inlay case, three design parameters, which were *L*_*i*_, *L*_*p*_, and *W*, were found with significant influence on the MPS in the enamel. Therefore, the influence of *L*_*i*_ and *L*_*p*_ on the MPS was evaluated under different levels of W, as shown in Fig. 4a–c. The peak MPS values were between 214.23 and 279.93 MPa, between 211.35 and 258.91 MPa, and between 196.55 and 234 MPa, respectively. The RMSE were 2.35%, 1.64%, and 1.55%, respectively. Three response surfaces of the peak MPS in the LD inlay for the three levels of *L*_*i*_ were shown in Fig. 4d–f, where the design parameters with significant influence became *L*_*p*_ and *D*_*i*_. The peak MPS values were between 269.2 and 317.27 MPa, between 271.56 and 310.4 MPa, and between 268.08 and 296.88 MPa. The RMSE were 4.21%, 2.70%, and 2.38%, respectively.

#### Peak ITS on the buccal side

**Fig. 5:**
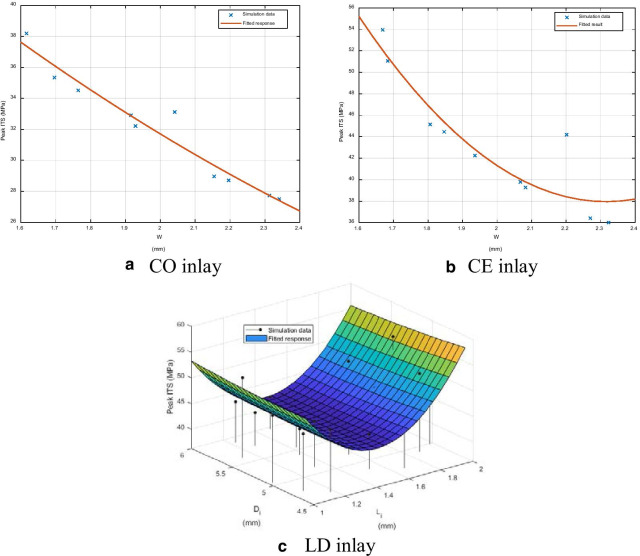
The fitted peak ITS responses in enamel on buccal side

For the CO inlay, *W* was the only high-impact parameter for the peak ITS on the buccal side of the enamel-inlay interface; therefore, the relation between *W* and the peak ITS was plotted, as seen in Fig. 5a. The ITS value was between 27.50 and 38.19 MPa, and the RMSE was 2.42%. Similarly, the peak ITS was affected by *W* for the case of CE inlay, as shown in Fig. 5b. The peak ITS was between 36.01 and 53.95 MPa with an RMSE of 15.95%.

For the LD inlay case, the peak ITS value on the buccal side of the enamel-inlay interface was between 62.55 and 67.05 MPa. The response surface corresponding to *L*_*i*_ and *D*_*i*_ was shown in Fig. 5c, with the RMSE of 1.30%.

#### Damage risk assessment

**Fig. 6:**
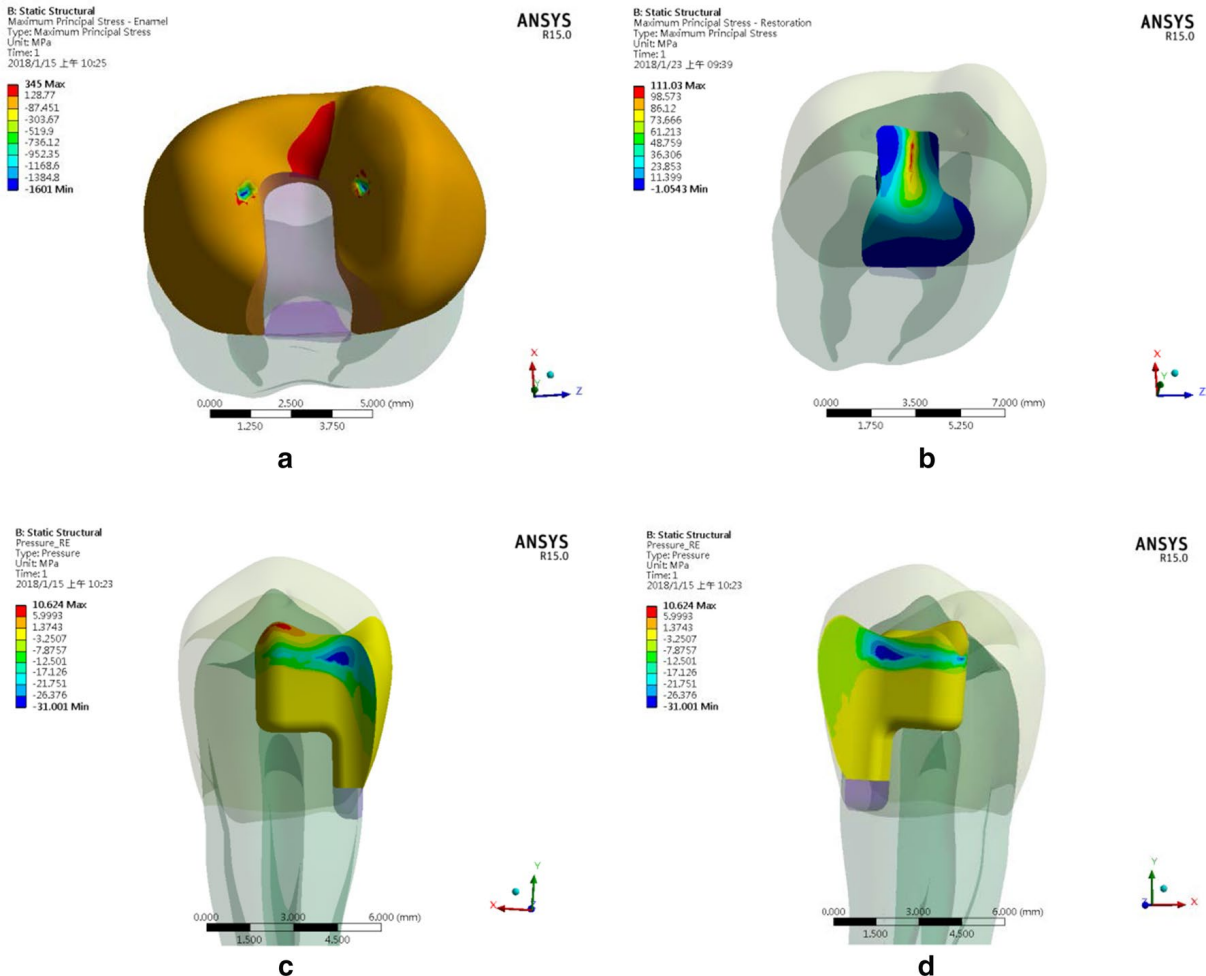
A typical case (CO inlay) of simulated MPS contours of enamel (**a**) and inlay (**b**), and ITS contours of inlay-tooth interface on lingual side (**c**) and buccal side (**d**)

When Young’s modulus of inlay material increased, the peak MPS in the enamel gradually declined. We also found that in every design point, the peak MPS occurred at the bottom of the occlusal valley, with a direction towards outside of tooth surface (A typical case, using CO inlay as an example, is shown in Fig. 6a, b). The tensile strength of the enamel was approximately 42.2 MPa under the load parallel to the enamel rod and was about 11.5 MPa under the load perpendicular to the enamel rod [31]. Because the arrangement of the enamel rod was perpendicular to the enamel surface, the peak MPS affected the weaker direction of the enamel structure in all three types of cases in this study and was much larger than the tensile strength of the enamel at this direction. The location and direction of the inlay’s peak MPS appeared the same as those of the enamel’s. Still, the peak MPS value of the inlay demonstrated a gradually increasing trend when the inlay material became stiffer. Particularly in the LD inlay case, the peak MPS in the inlay exceeded that in the enamel due to a large difference in material stiffness between the two materials.

The peak ITS on the buccal and lingual sides of the inlay-enamel interface for all inlay materials occurred at two axial walls of the isthmus and were slightly greater than the commonly seen adhesive strength of the dental adhesives for enamel (approximately 30–40 MPa) [31]. The peak ITS on the lingual side of the dentine interface declined slightly following the increase in Young’s modulus of the inlay material. The peak ITS on the buccal side retained near the strength of the adhesive for dentin bonding. The peak ITS on the two sides of the interface also occurred on two axial walls of the isthmus, which were close to the commonly observed adhesive strength of dental adhesives for dentin (approximately 20–25 MPa) [32, 33]. The ITS contour of the CO inlay and dentin interface is as displayed in Fig. 6c, d.

The values of stress indexes and corresponding fracture strength of all three cases are summarized in Table 1.

**Table 1:** Summary of peak stresses and corresponding fracture strengths

	MIN (MPa)	MAX (MPa)	Fracture strength (MPa)
CO inlay			
MPS in enamel	316.45	405.94	11.5
MPS in inlay	106.63	135.06	45
ITS (enamel-inlay, lingual)	32.05	37.54	40
ITS (enamel-inlay, buccal)	27.50	38.19	40
CE inlay			
MPS in enamel	251.36	306.16	11.5
MPS in inlay	189.11	218.77	42.5
ITS (enamel-inlay, lingual)	40.42	46.86	40
ITS (enamel-inlay, buccal)	36.01	53.95	40
LD inlay			
MPS in enamel	196.55	279.93	11.5
MPS in inlay	268.08	317.27	43.4
ITS (enamel-inlay, lingual)	45.48	63.13	40
ITS (enamel-inlay, buccal)	41.23	67.04	40

## Discussion

The finite element analysis has been used in many fields of dentistry [34]. With the advancement of 3D reconstruction technology, biomechanical evaluations can be performed on the more realistic human anatomy to significantly reduce animal and clinical trials. FEM-related studies are useful for clinicians to evaluate various prosthetic designs [28, 35, 36], however, only a few distinct designs can be considered in each study. The approach used in the present study, including automated modelling and response surface analysis, was able to comprehensively inspect the entire design space, to simultaneously identify the importance of each design parameter and to recognize the interaction between cavity shape and material selection.

### Main results and clinical relevance

Based on our study, we recommend that the isthmus be designed as wide as possible when using an LD inlay for restoration. A larger *W* could lower the peak MPS and reduce the peak ITS on the buccal side at the inlay-enamel interface to approximately 41 MPa, close to the maximum strength of clinically used dental adhesives. In terms of selecting the size of *L*_*i*_ to be configured with the larger *W*, a value between 1.5 and 1.6 mm can enhance mechanical performance.

From the mechanical standpoint, the results indicated that the use of the DME layer has little influence on the restoration quality and the risk of restoration failure. The MPS of the DME layer and the ITS at the bonding surfaces between the DME layer and other materials were much lower than their failure strengths. Also, the thickness of the DME layer did not exhibit a significant effect on any of the stress indexes evaluated in this study. These findings are consistent with the observations of the previous studies.

In summary, properly selecting the inlay material and specifying the geometric parameters could enhance the mechanical performance of the dental structures and reduce the volume of natural dentin removed.

### Detailed design guides for DME

For CO and CE inlays, the peak MPS of the enamel exceeded its failure strength the most among all stress indexes in our simulation under the loading condition like chewing hard substances, followed by the peak MPS in the inlay. For the peak ITS, a value higher than the adhesive strength was observed in the lingual and buccal sides of the inlay-enamel interface. The risk of failure caused by the peak MPS in the enamel and inlay is higher than that of the debonding failure. Therefore, the primary objective would be to reduce the peak MPS in the enamel followed by that in the inlay.

The peak MPS in the enamel exhibited a negative correlation with the two high-impact parameters, *L*_*i*_ and *L*_*p*_, enabling the magnitudes of variation to reach 85 and 55 MPa, respectively. However, when *L*_*i*_ was greater than 1.5 mm, or when *L*_*p*_ was greater than 2.6 mm, the peak MPS remained steady between the intervals of 310–330 MPa and 250–260 MPa, respectively. The peak MPS of the inlay exhibited a positive correlation with both *L*_*i*_ and *L*_*p*_, and the magnitude of variation of the peak value was approximately 30 MPa (variation range was 106.63–135.06 MPa for the CO inlay and 189.11–218.77 MPa for the CE inlay). The peak ITS at the enamel-inlay interface on the buccal side exhibited an almost linearly negative correlation with W, with the magnitudes of variation of the peak value being approximately 10.5 MPa and 18 MPa, respectively (variation range was 27.50–38.19 MPa for the resin inlay and 36.01–53.95 MPa for the ceramic inlay). Based on the previous observation, we recommend that while using CO and CE inlay, *L*_*i*_ and *L*_*p*_ should be shortened as much as possible. However, to ensure that the MPS of the inlay be reduced as much as possible while keeping the minimal peak MPS occurs in the enamel, *L*_*i*_ should not be less than 1.5 mm, and *L*_*p*_ should not be less than 2.6 mm. Finally, a larger *W* could effectively relieve the ITS on the buccal side of the enamel-inlay interface, such that the risk of debonding failure could be reduced.

For the LD inlay, the peak MPS in the inlay exceeded that in the enamel such that the inlay became the one with the highest risk of failure. The risk of debonding failure was similar on both sides of the inlay-enamel interface but was much lower than that of the peak MPS in the inlay and enamel. Therefore, the design strategy should focus on reducing the peak MPS in the inlay and enamel, and a higher priority should be placed on the former region.

The variation in the peak MPS of the enamel was similar in all three inlay cases, with all exhibiting a negative correlation with *L*_*i*_ and *L*_*p*_. The only difference was that for the LD inlay, *W* also demonstrated a significant influence on the peak MPS when W was less than 2 mm. The influence of *L*_*i*_ and *L*_*p*_ on the peak MPS for the LD inlay varied at different levels of *D*_*i*_ and was difficult to generalize. In summary, in the LD inlay case, the *W* of the cavity played a vital role in the mechanical performance of the tooth. When *W* increased, the peak MPS of the enamel significantly declined, and the peak ITS at the inlay-enamel interface also declined. *L*_*p*_, however, exhibited a significant negative correlation only with the peak MPS of the enamel; no significant interaction was observed with other stress indexes. *L*_*i*_ exhibited a significant upward, concave parabolic relationship with the peak ITS at the inlay-enamel interface on the buccal side of enamel when *W* was at its highest level (2.4 mm), and the smallest peak ITS could be obtained when *L*_*i*_ was 1.5 mm.

### Comparison with existing studies

With the advancement of dental composite and adhesive materials, marginal integrity of a restoration with DME can be comparable to those without DME [7–9]. For those cavities which require DME before placing the restoration, their tooth structures are usually compromised. The prosthetic consideration should prevent from further damages such as crack propagation or fractures. A recent study indicated that both inlay and onlay ceramic restorations are beneficial designs to prevent crack propagation [40]. Placing a material of low elastic modulus underneath the restorations such as another recommended design, gold crown with resin filling inside, could help absorb unfavorable stress to protect the remaining tooth structure. The DME layer in the present study plays the same role with low MPS and ITS to ensure the long-term integrity of structure and bonding interface.

The Onlay design can certainly provide better structural protection because of larger coverage [41]. To preserve natural tooth structure as much as possible, proximal or MOD inlay should always be first considered. Similar to our findings, the increased isthmus width of a MOD inlay can help reduce structural stresses [42]. Interestingly, the shear stresses were not increased with the increase of cavity depth. Relatively lower ITS was also found over the deeper DME layer in the present study.

### Strengths and limitations of the study

This study combined the FEM technique with design of experiments method to comprehensively investigate the entire design space of the DME problem. The automated modeling workflow allowed us to evaluate a large amount of finite element models with minimal manual work. The response surfaces, main effects, and analysis of variance helps clinicians to understand more detailed influences of the design parameters than traditional parametric studies. Moreover, to provide better clinical relevance, our numerical data was in vitro validated.

The simplifications of the finite element model and the in vitro experiment model developed in this study should be stated. First, the cement layer was not fully simulated. This study focused on the inlay design and assumed the cement layer would not fail. Secondly, the experimental setup of in vitro validation was simplified due to the manufacturing limitation. However, the validation test was successful and showed that our FE model was reasonably set.

## Conclusions

This paper evaluated the mechanical performance of DME technique for carious cavities. The finite element analysis combined with the design of experiments methods was used to investigate the class II inlay with various geometrical designs and different material types. Within the limitations of and assumptions made in this study, our investigations suggest that: (1) when CO or CE inlay is selected clinically, *L*_*i*_ and *L*_*p*_ should be shortened as much as possible; for simultaneously reducing the MPS of the inlay and maintaining the minimal peak MPS within the enamel, it is recommended that *L*_*i*_ and *L*_*p*_ are greater than 1.5 mm and 2.6 mm, respectively. Furthermore, enlarging *W* is recommended for lowering the risk of debonding failure; (2) when LD is used clinically, the width of the cavity would be the dominating factor for the mechanical performance of its restoration; (3) the thickness of the DME layer did not significantly influence either the structural stress or interfacial stress. As for the future perspectives, the integration of this biomechanics-based optimization approach with digital workflow of restorative dentistry should be developed. One should remain cautious while using DME clinically, this additional DME layer increases the number of bonding interfaces where failures usually start. Further fatigue analysis at the interface should be conducted to assess long-term stability.

## Supplementary Information

**Additional file 1.** The peak MPS in enamel, inlay, and DME layers (left to right) plotted under different levels of the design parameters in CO, CE, and LD inlays (top to bottom).

**Additional file 2.** The peak ITS of inlay to enamel on buccal and lingual sides (columns 1 and 2) and inlay to dentin on buccal and lingual sides (columns 3 and 4) under different levels of the design parameters (from top to bottom: CO, CE, and LD inlays).

## Data Availability

The datasets used and/or analyzed during the current study are available from the corresponding author on reasonable request.

## Abbreviations

DME: Deep margin elevation
PBE: Proximal box elevation
GIC: Glass ionomer cement
MOD: Mesial-occlusal-distal
CEJ: Cementoenamel junction
SEM: Scanning electron microscope
CO: Composite resin
CE: Ceramic
LD: Lithium disilicate
ITS: Interfacial tensile stress
MPS: Maximum principal stress
RMSE: Root-mean-square error
