# Supplementary material for: Investigating inlay designs of class II cavity with deep margin elevation using finite element method
Source: BMC Oral Health. 2021 May 16;21:264. doi: 10.1186/s12903-021-01630-z (PMC8127254; doi:10.1186/s12903-021-01630-z)

Additional file 1: The peak MPS in enamel, inlay and DME layers (left to right) plotted under different levels of the design parameters in CO, CE, and LD inlays (top to bottom).

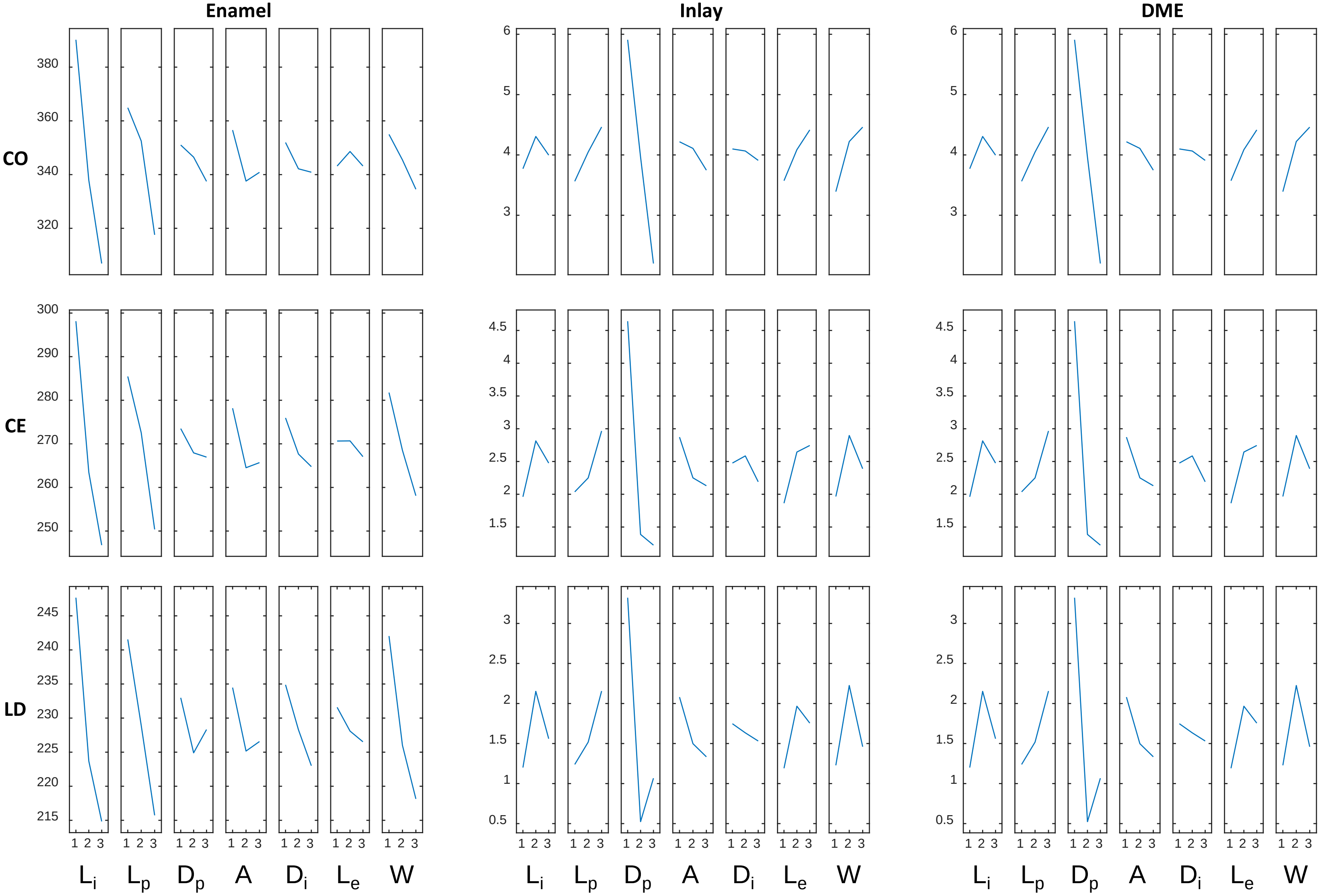

Supplement: Supplementary file 1 — Additional file 1. The peak MPS in enamel, inlay, and DME layers (left to right) plotted under different levels of the design parameters in CO, CE, and LD inlays (top to bottom). [file 12903_2021_1630_MOESM1_ESM.pdf]
