# Supplementary material for: Investigating inlay designs of class II cavity with deep margin elevation using finite element method
Source: BMC Oral Health. 2021 May 16;21:264. doi: 10.1186/s12903-021-01630-z (PMC8127254; doi:10.1186/s12903-021-01630-z)

Additional file 2: The peak ITS of inlay to enamel on buccal and lingual sides (columns 1 and 2) and inlay to dentin on buccal and lingual sides (columns 3 and 4) under different levels of the design parameters (from top to bottom: CO, CE, and LD inlays)

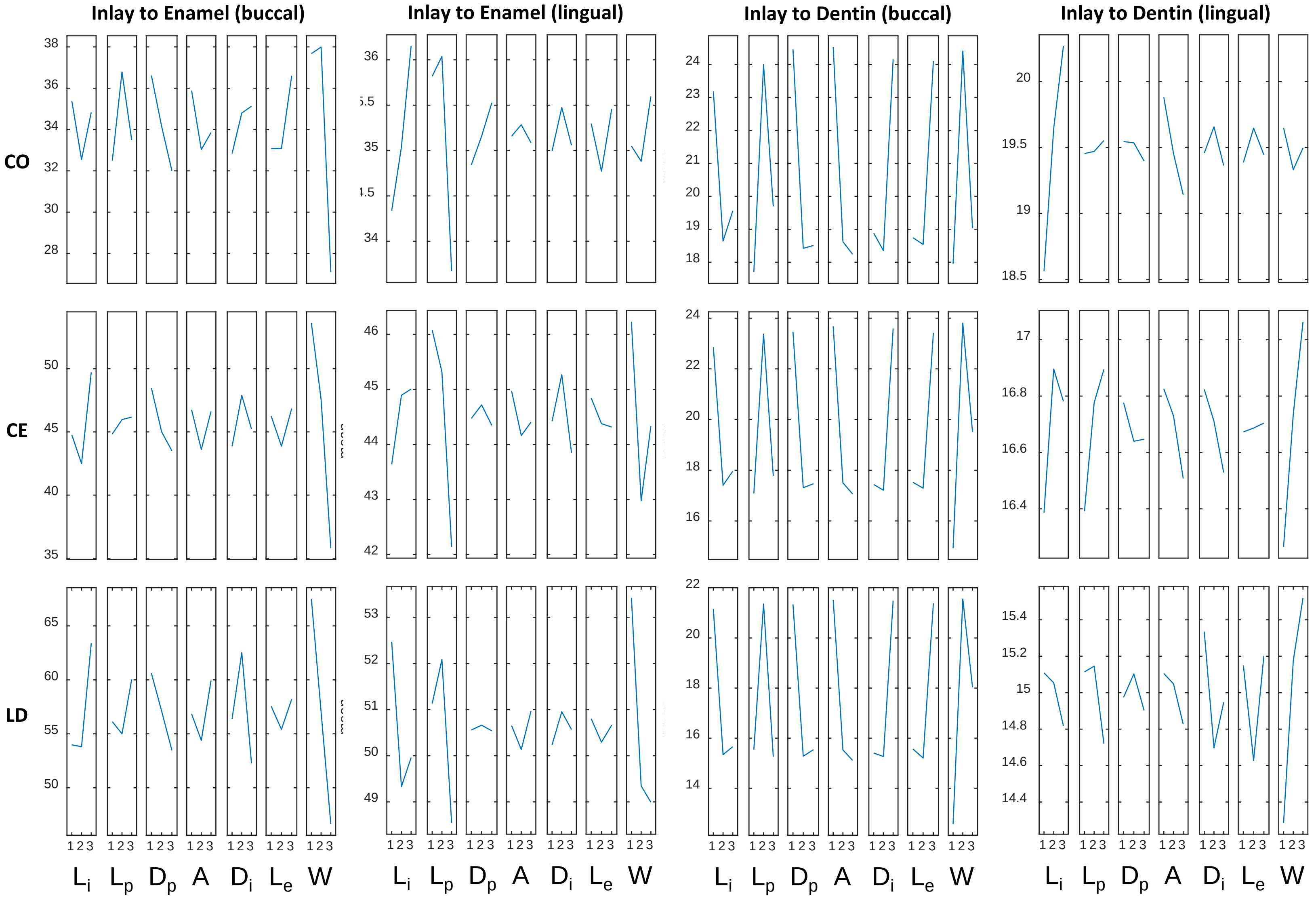

Supplement: Supplementary file 2 — Additional file 2. The peak ITS of inlay to enamel on buccal and lingual sides (columns 1 and 2) and inlay to dentin on buccal and lingual sides (columns 3 and 4) under different levels of the design parameters (from top to bottom: CO, CE, and LD inlays). [file 12903_2021_1630_MOESM2_ESM.pdf]
